# Almost Forgotten Research Contexts: William Stern’s Giftedness Research

**DOI:** 10.3390/jintelligence11090174

**Published:** 2023-08-29

**Authors:** Rebecca Heinemann

**Affiliations:** Department of Pedagogy, University of Augsburg, Universitätsstraße 10, 86159 Augsburg, Germany; rebecca.heinemann@gmx.de

**Keywords:** giftedness research, child psychology, personalism, philosophy, intelligence tests

## Abstract

This article examines the concept of intelligence and giftedness of the German psychologist and philosopher William Stern, the leading intelligence and giftedness researcher in Germany from the early 20th century to 1933. Stern developed a multifactorial giftedness model that integrated empirical and philosophical perspectives and was thus far ahead of his time. This concept was not taken up for a long time—not least because of the break that the research on giftedness suffered in Germany in 1933—and has not yet been presented with the required complexity and interdisciplinarity. In the USA, Stern’s research has so far been reduced to the IQ formula he created. The author presents Stern’s concept of giftedness in the context of the particular scientific–historical and educational–political situation in Germany in the first third of the 20th century. The pedagogical conclusions that Stern associated with the research on giftedness, and which essentially referred to the requirement to support all gifted children, regardless of social class, are also illuminated.

## 1. Introduction

William Stern (1871–1938) was the most important representative of talent and giftedness research in Germany, which got underway internationally in the first decade after the turn of the century. Inspired by French test research, he and his students conducted large-scale intelligence surveys in schools. On this basis, the intelligence quotient was created, which Stern first presented in 1912, in his book “*Die psychologischen Methoden der Intelligenzprüfung und ihre Anwendung an Schulkindern*” (“The psychological methods of intelligence testing and their application to school children”; Stern 1912). His research was often reduced to this IQ formula, especially in the USA (Lamiell 1996). It was and is overlooked that William Stern’s concept of talent was not only empirically based. Rather, the consistent combination of empirical and philosophical foundations was characteristic. Only by considering both sides together it is possible to adequately present Stern’s complex, multi-dimensional concept of talent. Through the philosophy of critical personalism, which he developed from 1900 onward and finally presented in three comprehensive volumes (Stern 1906, 1918c, 1924), Stern combined empirical studies of talent psychology with a philosophically grounded theory of talent. It focuses on the psychological orientation towards human individuality and individual talents.

The personalistic basic assumptions form the cornerstones of the scientific conception of talent, which understands talents as valuable and meaningful dispositions and accentuates proactivity, personal self-determination, and the human being’s options for creative design.

Unlike his contemporary Lewis M. Terman, who had a decisive influence on giftedness research in the USA, William Stern’s person and work are far less well-known today. Various reasons must be discussed to explain why this congenial German scientist has been forgotten for decades.

The National Socialist seizure of power in 1933 marked a significant turning point for talent and giftedness research in Germany, and its impact can hardly be overestimated. Research into giftedness and societal interest in the gifted came to a standstill. Unlike the first German democracy, which was installed in 1918/1919, the Nazi regime negated children’s rights to education. This put an end to the initiatives of the first democratic state, teachers’ associations, and scientific institutes in this field. From then on, it was not the children’s individual talents that formed the guideline but the National Socialist state’s claim to power and its interest in instrumental “selection” (“Auslese”). Added to this was the dismissal of scientists who had worked in the field of intelligence and talent research and thought psychology during the Weimar Republic. Like William Stern, who was born in Berlin in 1871, many of these scientists had a Jewish background and were dismissed from civil service and universities as early as in 1933, due to the anti-Semitic legislation of the Nazi regime. Stern emigrated to the USA in 1934, where he taught at Duke University until his death in 1938. There, what he himself regarded as the core of his work, the philosophy of personalism, was met with disinterest.^1^ In Germany, decades would pass before individual German psychologists dealing with the history of their discipline “rediscovered” the work of William Stern: the focus here was on differential and child psychology, but not on talent research^2^, which Stern himself considered to be one of his most important research areas.

The following essay presents William Stern’s concept of talent by the required complexity and interdisciplinarity and against the background of contemporary scientific, social, and school historical contexts. An attempt is made to compare German and American talent research. Similarities and differences are worked out by the example of William Stern and Lewis Terman. 

## 2. William Stern’s Personalistic Concept of Intelligence and Giftedness

### 2.1. Scientific–Historical Background: Observations of Children

Firstly, the scientific–historical contexts of Stern’s early talent research should be considered. William Stern’s extensive child psychological studies are to be regarded as the central source of inspiration for the development of the concepts of “intelligence” and “talent”, which identify him as the most important representative of emerging child psychology in Germany. Methodologically, he leaned heavily on American research, which at an earlier stage, namely by the end of the 19th century, worked with so-called baby diaries, with close caregivers, usually the parents, documenting the development of small children in diaries. William Stern and his wife Clara (1877–1945) undertook an extensive diary project that began with the birth of their first child, Hilde (1900–1961). The couple examined the language development, play, design, discovery, fantasy, feelings, memory, and testimony, as well as aesthetics, values, and ethics of their children from birth to adolescence over a period of time which was longer than the previously common investigation period of the child’s first three years of life. Günther was born in 1902, and Eva in 1904. The detailed observations, which fill almost 5000 manuscript pages, formed the basis for the first child psychological monographs by Clara and William Stern, such as their jointly authored studies “Die Kindersprache” (“Children’s speech”, Stern and Stern 1907) and “Erinnerung, Aussage und Lüge in der frühen Kindheit” (“Recollection, testimony, and lying in early childhood”; Stern and Stern 1909), as well as the book “Psychologie der frühen Kindheit bis zum sechsten Lebensjahre” (“Psychology of early childhood up to the sixth year of life”, Stern 1914a), published by William Stern in 1914, which became a standard work of child psychology in Germany. Research has so far ignored the fact that the observation of children’s thinking, intelligence, and individual talents also played a central role in the diary entries (Heinemann 2023). This can be observed to a greater extent around 1905, when Alfred Binet presented his first, internationally acclaimed intelligence test (Funke 2006). Scientifically, William Stern regarded early childhood as the decisive phase for the development of intelligence and talent.

These processes were influenced by the family and a stimulating environment for the child. Considerable weight was attached to genetic factors, although these, as Stern emphasized, did not determine the development.

The couple’s pronounced interest in the different talents of children, which also attached great importance to creative processes (drawing, composing, writing), can also be seen in the context of the contemporary progressive pedagogy, which focused on the individual talents of children. This emphasis was combined with a massive criticism of the contemporary school system, which disregarded the individuality of the pupils. Clara and William Stern articulated this criticism in massive form. They associated this with the demand for an individualization of education and instruction, for the promotion of intellectual interests, independence, and spontaneity, and the ability of children to express themselves.

Clara and William Stern particularly emphasized the different talents of their three children. William Stern himself considered giftedness research to be the most important topic in the differential psychology that he systematically developed after 1900 (Stern 1911a). By his concept of individuality, he linked the ethical postulate of respecting the uniqueness of children’s talents and promoting them accordingly.

### 2.2. Philosophy of Critical Personalism as a Theoretical Basis

Observations of children were also the starting point for the development of critical personalism, as Stern himself writes (Stern 1927). This philosophical system focused on the concept of “person” (“Person”) and distinguished it from “thing” (“Sache”). In relation to the human person, its uniqueness (singularity), self-worth, meaningfulness, goal-directedness, and proactivity are worked out. The development of human intelligence and talents, which were to be promoted as meaningful and valuable “dispositions” (“Anlagen”), are understood as dynamic processes that are caused by a close meshing of endogenous and exogenous factors, as well as the “personality” itself, which is understood as the originator and actor of development processes (Stern 1918a, 1918c). In the triangularity of development processes, for which Stern used the central term “convergence”, he countered what he saw as the reductionist approaches of experimental pedagogy and behaviorist psychology. In contrast to the experimental psychology he criticized, Stern emphasized the impossibility of comprehending a system solely from parts and their mutual relationships. Personalism understands mental and physical processes as a unit: as a person, the human being always acts and reacts as a whole.

The epistemological implications of Stern’s system of thought are methodologically significant. Stern’s aim was to overcome the contradiction between scientific psychology and philosophy. In this context, he emphasized the connection between empirical–experimental and philosophical approaches, which are not mutually exclusive but complement each other. With the anthropological concept of the person, which gave expression to the biological dimension and conditionality and the self-causality, spontaneity, and the end in itself, Stern associated the demand for a methodically pluralistic concept that integrated nomothetic procedures and ideographic, individualizing methods. This approach is of the utmost importance for Stern’s intelligence and talent diagnostics. It characteristically integrates experimental methods, such as the use of intelligence tests and observation methods, including qualitative analysis, e.g., of drawings, poems, or compositions by children.

### 2.3. Differences between US and German Intelligence Research—Dealing with Intelligence Tests

A crucial difference between German and American talent research—this can already be stated for its early history in the first decades after 1900—was the scientific conception of intelligence tests and their application. If one compares how the concepts of “intelligence” and “talent” are dealt with, on the one hand, and intelligence tests on the other, there are crucial differences between the USA and Germany. While there were early signs of acceptance of the concept of intelligence and of intelligence tests in the USA, there were many more reservations in Germany (Kössler 2016; Ingenkamp 1990). The publication of the Stanford Revision of the Binet Tests by Lewis Terman (Terman 1916) marked a major turning point in the history of the American testing movement. The new Stanford-Binet test subsequently became a standard tool of clinical psychology, psychiatry, and school counseling. It is true that some American scientists also voiced concerns about the tests, which in their view were vastly overestimated (Carson 2007). The psychologist Edward L. Thorndike (1874–1949) pointed out that intellectual abilities were context-dependent. The educator and philosopher John Dewey (1859–1952) also criticized the assumption that people could be classified “correctly” by intelligence tests. Nevertheless, numerous psychologists were enthusiastic about the newly developed methods of measuring intelligence and the prospect of being able to identify not only the weak and the highly gifted but all students, and to allocate them to the “appropriate” type of school. However, tests were not only used in schools. In contrast to Germany, there was a broadening of the tests, which were considered to have a strong socio-political relevance that went far beyond the school framework. It was assumed that immigration could be regulated with the help of test research. At Ellis Island, intelligence tests were used to identify newly arrived immigrants of “below-average intelligence”, in the context of which clearly racist implications became apparent (Isensee 2021; Carson 2007). Intelligence tests were first used en masse in the US Army during World War I (Chapman 1988).^3^ In general, there were significant differences between American and German academic traditions. In contrast to the Anglo-American world, the prevailing concept of science in Germany, which assumed a dichotomous relationship between the scientific and non-scientific areas and postulated that science was “purposeless” (“zweckfrei”), tended to oppose the application of scientific knowledge. This is clearly reflected by the orientation of the disciplines of education and psychology.^4^

First and foremost, however, the assumption that clearly prevails among American and British scientists must be stressed, that intelligence is a genetic and unchangeable quantity that not only differs from person to person, but characteristically, differences in intelligence between social groups and ethnic groups (“races”) were declared to be “natural”, which served for stabilizing the existing social order. The use of a static concept of intelligence clearly distinguished Anglo-American research from Alfred Binet (1857–1911), whose “first” intelligence test, published in 1905, was received, translated, and “imported” internationally. Binet was strongly influenced by the thoughts of John Stuart Mill (1806–1873) and assumed that intelligence could be influenced (Freitag 2014; Funke 2006). In his view, intelligence tests also represented a means of determining a child’s individual educational needs (Baudson 2014; Lewontin et al. 1988). Another feature of the American testing movement was its association with eugenics and sterilization laws targeting criminals, “idiots”, sex offenders, alcoholics, or drug addicts (Carson 2007; Lewontin et al. 1988). Eugenics, which assumed a “higher” and “lower” value of human beings, was closely linked to talent research and the testing movement from the start. Eugenic thinking was very pronounced in Terman’s work, for example, who was a member of several pro-eugenic organizations such as the Human Betterment Foundation, and associated his talent research with recommendations on how to deal with people who were tested to show “below-average intelligence” (Isensee 2021).

In Germany, it was initially mainly representatives of experimental pedagogy who underscored the advantages of intelligence tests. In their view, the tests placed student assessments on an objective basis and removed them from the subjective judgment and arbitrariness of teachers. Against the background of a school criticism that focused on the school of the German empire as an anonymous mass enterprise, progressive educators favored the use of exact diagnostic procedures which were supposed to supplement the traditional teacher’s judgement. On an experimental basis, over- and under-challenging of students could also be avoided, and targeted support could become possible. There was a close connection between experimental test research and contemporary progressive pedagogy, which is also reflected by the membership of numerous supporters of test research in international progressive pedagogical organizations such as the New Education Fellowship (Ydesen 2011). The founder of experimental education in Germany, Ernst Meumann (1862–1915), was the first German scientist to deal with the French talent research. In the first decade after the turn of the century, Stern also intensively dealt with the Binet test, which examined the memory performance, visuality, imagination, attention, suggestibility, understanding, aesthetic sensitivity, willpower, moral awareness, motor skills, and spatial perception of children and further developed Binet’s approach. What “intelligence” is and what constitutes “intelligent” behavior were central questions that Clara and William Stern had been working on in their development diaries since around 1905 (see above; Heinemann 2023). Initially—it should be noted—Stern assessed Binet’s test procedure negatively. In 1897, he wrote to his friend from college, the Freiburg philosopher Jonas Cohn (1869–1947): “Of course, Binet with his ‘mental tests’^5^ comes off very badly for me” (Lück and Löwisch 1994, p. 24). In the central work “Die Differentielle Psychologie in ihren methodischen Grundlagen” (“Differential psychology in its methodological foundations”), which was published a few years later and contains a detailed section on the development of intelligence tests (Stern 1911a, p. 92 ff.), Stern then emphasized “the inestimable importance” (ibid., p. 101) of Binet’s test. However, at the same time, he criticized the too-rough specification of the value which is determined by subtracting age from the intelligence age. The publication was preceded by a comprehensive critical review and further development of the Binet test at Schlesische Friedrich-Wilhelms-Universität by Stern and his colleagues. Characteristic of Stern’s working method was the interdisciplinary cooperation between scientists, psychologists, doctors, and teachers. The Breslau chemist Otto Bobertag (1879–1934) translated the Binet test into German and compiled a complete overview of Binet’s work (Bobertag 1909). Stern used the Binet test to conduct intelligence studies at Silesian schools and discussed the methodology and application of intelligence tests in university courses (Heinemann 2016). Numerous further training courses for Silesian teachers’ associations also bore witness to his goal of familiarizing teachers with the new intelligence diagnostic. His pronounced commitment to the further training of teachers was combined with the openness of this professional group towards school organizational and pedagogical reforms and the interest in further scientific training. In addition to new scientific findings in child and adolescent psychology, the lecture cycles and courses organized by Stern enabled the participating teachers to intensively deal with talent diagnostics and the methods of intelligence testing (Heinemann 2016). A year after the publication of the work “Die Differentielle Psychologie”, which served for classifying the topic in a systematic scientific manner, Stern discussed the application of differential research in his book “*Die psychologischen Methoden der Intelligenzprüfung und ihre Anwendung an Schulkindern*” (Stern 1912). The work was published in the USA two years later, translated by the American psychologist Guy Montrose Whipple (1876–1941) under the title “*The Psychological Methods of Testing Intelligence*” (Stern 1914b). It was the first overall presentation in which Stern presented his concept of intelligence and the IQ formula and critically discussed international research and the use of intelligence tests. He formulated his classical teleological concept of intelligence: “Intelligence is the general ability of an individual to consciously adapt his thinking to new demands; it is general intellectual adaptability to new tasks and conditions of life” (Stern 1912, p. 3). Stern judged the use of tests in schools to be fruitful. In contrast to the common pedagogical examinations, which tested knowledge and relied on external performance effects, the tests could provide “an index of the child’s inner disposition, its mental maturity and ability” (Stern 1912, p. 7). However, the intellectual advantage or deficit with younger children compared to older children had to be expressed mathematically. In this context, Stern introduced the “intelligence quotient”, which shows the ratio of a child’s intelligence age to their age and is obtained by dividing the intelligence age by the age.^6^

### 2.4. Stern’s Criticism of Intelligence Tests

From the outset, Stern associated this scientific conception with a warning against any premature and uncritical application of the tests. He expressly referred to the use of intelligence tests in the USA, which, for example, in the state of New Jersey, were mandatory for all children who could be considered mentally retarded. He judged this to be a failure. The assumption of the “ease” of the test application was also a mistake. Rather, the tests required a high level of professionalism and practice and required a “critical mind” (Stern 1912, p. 9).^7^ In addition, Stern warned against overestimating the test results. These represent “at best the psychographic minimum [that] allows for a first orientation with individuals that one otherwise does not know at all” (ibid.). According to Stern, testing the intelligence of children required an interdisciplinary approach and the cooperation of psychologists, pedagogues, and pediatricians.

Stern’s positioning should also be viewed in the context of the talent debates that took place in Germany in the early 20th century. At the early interdisciplinary congresses of child psychology, many pedagogues expressed skepticism about talent research. At the congress of the reform-pedagogical “Bund für Schulreform” in Dresden in 1911, which dealt primarily with questions of intelligence and talent, congress participants warned of the danger of “one-sided intellectualism” (Bund für Schulreform 1912, p. 32). Representatives of contemporary progressive pedagogy, who massively criticized the contemporary school system, wanted to avoid this impression. With regard to a certain degree of “one-sidedness” in the use of intelligence tests in the USA, the progressive pedagogue Hugo Gaudig (1860–1923) positively emphasized Stern’s “level-headed” treatment of the subject (Bund für Schulreform 1912, p. 33). Of note to be further considered is the predominantly philosophical–humanities orientation of German pedagogy after the turn of the century.^8^

At the end of the 1920s, Stern’s criticism of a methodologically un-reflected use of tests, which he observed primarily in the USA, intensified. Stern summed up the experiences of his second trip to the USA, which he had made in 1928 for the International Psychological Congress in New Haven:

“To a much greater extent than the laboratory experiment, the outward appearance of American psychology is determined by the method of the test. Since the war, in which the entire American army was tested for intelligence by a simple calibrated mass procedure, the testing method has reached an amazing—at times almost frightening—expansion. We find on the one hand the method of age grading introduced by Binet in the treatises of Terman, Kuhlman and others; When I introduced the term “intelligence quotient” 17 years ago as a measuring principle for such intelligence tests, I had no idea that the “I.–Q.” (pronounced: Ei-kjuh) had become a kind of everyday formula and one of the most common words in psychological technical language in America.” (Stern 1930a, p. 50)

### 2.5. Connection of Stern’s Giftedness Research and School Reform

In addition to his criticism of tests, other distinctive features of Stern’s talent and giftedness research that set him apart from mainstream US research must be taken into account. An emancipatory leitmotif was characteristic of Stern’s work, which stood for the possibilities of social advancement for children who were largely excluded from higher education in the German Empire and the Weimar Republic. Characteristic of the school system of the German Empire was the separate schooling of children from well-to-do bourgeois families, who attended three-year private preschools, which automatically made it possible to transfer to a higher school, and children of workers, craftsmen, farmers, or small tradespeople who, due to the socioeconomic status of their families, were restricted to a simple elementary school education. Stern advocated overcoming the strict dichotomy and demanded the greater permeability of the traditional school system of the 19th century, which around 1900 only gave about 3% of a year group the opportunity to attend a higher school (Gymnasium, Realgymnasium, Oberrealschule). In this context, he called for the targeted promotion of gifted “elementary school students” for whom “every path should be paved pedagogically” (Stern 1916b, p. 112). He certainly had in mind the experiences of the German Jews, who in the 19th century had achieved social advancement through education in a unique way (Lässig 2004)—the proportion of Jewish students at the higher schools of the Kaiserreich was about five times as high as that of children from Christian families (Schatzker 1988). Like William Stern’s parents, who were not well-off and paid for their gifted son to attend the renowned Köllnisches Gymnasium in Berlin, many Jewish families made great efforts to enable their children to obtain higher education. According to Stern, in the interests of individuals and the common good, society could not do without the potential of many gifted elementary school students. In this context, he pointed out the developability of intelligence: Children from the lower social classes, who lacked a stimulating environment at their parental homes, developed more slowly, and were sometimes at a disadvantage in terms of intellectual development compared to children from the middle class, who experienced a variety of stimuli from the family environment and whose mental development and intellectual performance were supported by conversations, books, games, or family trips. In this context, however, Stern emphasized that disadvantaged children could attain the same level of intelligence at a later point in time (Stern 1916a).

Contrary to what Schmidt assumed (Schmidt 1994), Stern was not in favor of the so-called “uniform school” (“Einheitsschule”), which was above all advocated by teachers of a social-democratic orientation in the Kaiserreich. Stern rejected the concept of the “Einheitsschule”, which envisaged the four-year joint schooling of “preschoolers” and “elementary school children” (Stern 1913), because he saw it as a disadvantage for the “preschoolers”, who would lose a whole year. Comprehensive social reforms were needed to eliminate existing disadvantages for working-class children. Other forms of school organization had to be found, e.g., separate courses that prepared gifted elementary school students for high school. In the Weimar Republic, Stern rejected the eight-year schooling^9^ demanded by social democrats and socialist education politicians. He pointed out that the omission of talent-differentiating lessons damages the personality and school performance development of gifted students, who needed special support measures and intellectual stimulation.

### 2.6. Critical Attitude towards Eugenics

Another distinguishing feature is Stern’s critical attitude towards eugenics. Stern emphasized that the nativistic conceptions of intelligence of eugenic concepts were at odds with the convergence theory of personalism. Against several representatives of eugenics, he argued that the dynamic concept of intelligence he advocated was not compatible with hereditary biological interpretations, according to which intelligence was a static variable that could hardly be influenced by the environment. The physician Fritz Lenz (1887–1976), who had held the first chair of “racial hygiene” (“Rassenhygiene”) in Munich since 1923, and Wilhelm Hartnacke (1878–1952), the Dresden city school board and minister of education in Saxony from 1933 to 1935, supported what Stern saw as a wrong assumption of biologically determined “inferiority” of dispositions in the lower social classes (Stern 1928a, 1930b). This criticism is also to be emphasized against the background of the increasing importance of eugenics in Germany, which in the Weimar Republic was by no means limited to the group of völkisch nationalists, but was widespread and, e.g., also “seeped” into the social democratic and catholic milieu. With a view to hereditary biological interpretations and the associated assumptions of a predisposition determination, Stern had already formulated in his 1918 publication “*Die menschliche Persönlichkeit*” (“*The Human Personality*”): “The singularity of the personality slips through the tightest net of heredity rules and because of this it can neither theoretically nor practically be cultivated!” (Stern 1918c, p. 115). In the revised second edition of the book “*Die Intelligenz der Kinder und Jugendlichen*” (Stern 1916a), he pointed out wrong data and calculation errors in the work of the American psychologist and eugenicist Henry Goddard (1866–1957), who assumed too-large a number of “less intelligent” children and an extraordinarily small number of children in the lower social classes who showed above-average intelligence.

In this context, it should be added that Stern’s ethics of talent underlined that a one-sided selection oriented towards economic considerations, which disregarded the personal significance and meaningfulness of individual talents, was ethically wrong (Stern 1924). In this context, Stern clearly criticized the personnel selection and a scientific and economic system that disregarded the person.

### 2.7. Stern and Terman: Similarities and Differences

Finally, the similarities and differences between Stern’s and Terman’s giftedness research (Minton 1988) are highlighted here. Stern showed great interest in Terman’s giftedness research. He defined the systematic research of all forms of giftedness and their promotion as the crucial task of differential child and youth psychology (Stern 1910; this central text was published one year later in the American Journal of Educational Psychology: Stern 1911b). In doing so, he himself pointed the way to the new research into the highly gifted. Stern initiated the first studies about gifted children in Germany. The “Institut für angewandte Psychologie und psychologische Sammelforschung” (“Institute for applied psychology and collective psychological research”), founded by Stern and his companion Otto Lipmann (1880–1933) in Berlin in 1906, and the associated institute journal formed the institutional framework for the first studies on gifted children, which were individual case studies (Stumpf 1908; Von Hornbostel 1910). When dealing with Terman’s studies on giftedness (Terman 1916, 1919), Stern also clearly had in mind the better financial resources of American universities and psychological research institutes, which made it possible to carry out larger-scale studies that could not be carried out in this form in Germany. In his report on his second research trip to the USA in 1928, Stern mentioned that he would have liked to have met Terman personally, but this was probably not possible due to time constraints (Stern 1930a). Important clues to Stern’s attitude towards Terman are provided in the final chapter of the revised fourth edition of the book “*Die Intelligenz der Kinder und Jugendlichen*”, published in 1928, where Stern discussed Terman’s study of giftedness, “Genetic studies of genius” (Terman 1925a). In the same year, a German translation of an article by Terman entitled “The nurturing of talent” appeared in the “*Journal for Educational Psychology and Experimental Education*”, co-edited by Stern, the leading German specialist publication in the field of child and adolescent psychology (Terman 1925b). Stern summarized that Terman provided important impetus for the research into the gifted. He positively emphasized the “very instructive” findings (Stern 1928a, p. 460) of the study, among which he counted Terman’s revision of the disharmony thesis. This widespread and already controversial thesis at the beginning of the 20th century, which assumed a connection between giftedness and a problematic, unstable, “disharmonious” personality development (Steinmetzer and Müller 2008), was clearly refuted by Terman (Stern 1928a, p. 461f.).^10^

At the same time. Stern articulated clear criticism. The study shows “one-sidedness” (ibid., p. 459, also for the following literal quotation). Above all, Stern criticized Terman’s omission of “individualizing deepening and analysis”. It is objectionable that the final selection of the examined gifted children was based exclusively on the test examination: “One concern cannot be suppressed here: because the final selection is based solely on the test examination, the ‘reactive’ intelligence is tested too one-sidedly. It is questionable whether the spontaneous, the creative, the one-sided talents are sufficiently included by such a kind of selection” (ibid., p. 460). Stern also described the title of the book as “misleading”: “Ingenious children will never be able to be read out by intelligence tests” (ibid.), he underlined, referring to his distinction between “genius” and “intelligence”.

In this context, the following should be added: William Stern basically assumed a “g-factor”^11^ in Spearman’s sense, a general level of human intelligence that could be experimentally determined (Stern 1928b). Genetic influences also play a significant role in Stern’s concept of intelligence, and one that should by no means be underestimated. The development diaries of Clara and William Stern, in particular, make this clear. However, there is one big BUT to add: Unlike Terman or Goddard, who stood in the tradition of Francis Galton (1822–1911) and understood intelligence as an inherited, static quantity, Stern worked out the developability and malleability of intelligence. According to Stern, intelligence was not an isolated quantity but needed to always be understood in the context of the unity of the person:

“Intelligence is neither a rigid, completely uniform faculty, nor is it a mere aggregate of “intelligences” that can coexist in an individual in any degree. Rather, it is the overall level of a personality, which does not represent a straight level, but experiences a qualitative, unique modeling in each person through crests and troughs.” (Stern 1928b, p. 2; quotation marks in the original).

Consequently, Stern and Terman judged the validity and importance of intelligence tests differently. In contrast to Terman, who in the 1920s had schoolchildren assigned to one of the five grades (“tracks”) of the progressive elementary school on the basis of tests (Chapman 1988) which assumed an exact fit between measured intelligence and grade choice, Stern emphasized that intelligence tests could only flank the assignment of a child to a certain type of school. He pointed out that “the IP [Intelligence test, RH] alone cannot at all determine how many and which children are to be transferred from elementary schools to higher schools; it can at most support these measures. One must never forget that intelligence is only a partial factor of higher performance, in addition to which special talents and, above all, willpower play an essential role” (Stern 1916a, p. 133).

According to Stern, a high intelligence quotient was a crucial feature of giftedness, but it was not the only criterion.^12^ Willpower, motivation, and commitment (commitment to a task) had to be added so that giftedness could develop. With this multifactorial giftedness concept, which can be compared to the giftedness model developed by Joseph Renzulli in the 1970s, Stern was decades ahead of his time.^13^ Further striking differences between Stern and Terman are worth noting. Lewis Terman’s handling of tests showed a strong interest in absolutely “serious” answers and a strongly adapted behavior of the tested children. Creative responses received only a low score on the Stanford-Binet test. For example, testers were instructed by Terman to count as “false” answers given by five-year-old children “for fun”. Here is how Terman explained using an example where the child had to choose the “prettiest” of two pictures: “Sometimes the child laughingly designates the ugly picture as the prettier, yet shows by his amused expression that he is probably conscious of its peculiarity or absurdity. In such cases “pretty” seems to be given the meaning of “funny” or “amusing”. Nevertheless, we score this response as failure, since it betokens a rather infantile tolerance of ugliness” (quoted after Carson 2007, p. 189).

The fact that four-year-old children answered “scream” when asked what to do when they were hungry was also rated as “wrong”. Terman insisted that the only correct answer had to be “food”. Elements such as “obedience” flowed into the tests. A sense of order, convention, knowledge, and “virtuous” behavior were expressed by a number, the IQ.

In contrast, Stern attached great importance to creative processes. He expressly pointed out the importance of highly gifted creative people who could also fall through the school grid (Stern 1910). Stern wanted the factor of creativity to be taken into account when evaluating intelligence and ability tests. He pointed out the importance of children’s thought processes and emphasized that an answer that was “wrong” in the sense of the examiner could also have a good meaning in the context of the child’s thought structure, and for this reason had to be judged as “correct”. For the child psychologist, it is often not the “correct” answer to a question that is of interest but the solution the child had found. Such an approach relied not only on testing but on qualitative analysis and extensive observation. A differentiated assessment of pupils is also evident in the pupil selection process conceived and implemented by Stern and co-workers for the first time by the end of the First World War, by means of which talented elementary school pupils were tested for their suitability for higher school education (Stern 1918b). Stern integrated the observation method in a characteristic way. A particularly careful but slower way of working in a child, as well as “strong independent thinking, meaningful questions and the ability to grasp essentials” (Stern and Peter 1919, p. 21), as determined by the teacher’s observation, could compensate for a negative test result.

## 3. Conclusions

William Stern developed a complex concept of talent that was hardly taken up in the USA in the interwar period. The different orientations of German and American talent and giftedness research can certainly also be seen as a crucial reason why relevant works by Stern were no longer translated into English in the interwar period, in contrast to the pre-war period (Stern 1910, 1912).^14^ Important reasons for the lack of discussion by German scientists concerning Stern are the break of 1933, and the lack of discussion by psychologists about the history of their discipline under National Socialism, which only gradually began in the 1980s. During the Nazi era, Stern’s books were banned and removed from German libraries. German psychologists, most of whom had willingly joined the Nazi regime in 1933, continued to set the tone in the post-war period. Only by the end of the 1950s—combined with the generation change—did a reorientation of psychology in the Federal Republic take place.^15^ To this is added the skeptical attitude that prevailed for a long time in the post-war society of the Federal Republic of Germany towards research and promotion of the highly gifted, which was under the general suspicion of pursuing elitist, anti-democratic motives. German giftedness research, which only experienced a boom at the beginning of the 1980s, was familiar with American giftedness research, but not with its own academic traditions, which had been largely founded by William Stern. The currently high topicality of this concept of talent, especially for a person- and value-oriented promotion of talent, gives hope for a further scientific debate on William Stern.

## Data Availability

Not applicable.

## Notes

1 A significant reason for Stern’s lack of reception in the USA is the different understandings of science. Stern criticized the exclusively scientific orientation of psychology and called for the combination of scientific and humanistic methods. In particular, he criticized behaviorist psychology, which in his eyes did not focus on human freedom and self-determination. An exception among American psychologists was the personality psychologist Allport (1938, 1968), who studied Stern’s work. More recent works that reflect Stern’s work come from Kreppner (1992) and Lamiell (2010, 2022).

2 German giftedness research was only “revived” in the 1980s and was initially still strongly focused on by US research. Due to the experiences of the Nazi era, for decades it had been associated with anti-democratic elitism in Germany. The early history of German talent and giftedness research in the first third of the 20th century was largely unknown at that time. After the Second World War, a middle-class society critical of the elite developed in the Federal Republic of Germany, most of which were opposed to the promotion of highly gifted people (Bergold 2013). The notion that broad funding came at the expense of promoting an already “privileged” elite was widespread up until the 1980s).

3 This contributed to the provisions of the Immigration Restriction Act of 1924, which limited the annual immigration of migrants to the United States to 2% of the proportion already immigrating to the United States from each affected country. The responsible politicians referred to Robert M. Yerkes’ intelligence studies on soldiers and derived from his results that immigrant soldiers were less intelligent, which was ultimately generally transferred to the group of immigrants. As a consequence, it was proposed that immigration from Asia, especially from China and Japan, should stop altogether, while immigration from southern and eastern European countries should be severely restricted.

4 The teaching of child psychological knowledge was already an integral part of teacher training in the USA by the end of the 19th century (Heinemann 2016).

5 The term “mental tests” comes from the Wundt student James McKeen Cattell, whom Binet was actually critical of.

6 Lewis Terman adopted Stern’s concept of the intelligence quotient and a few years later presented the Stanford-Binet test, which provided for multiplying IQ by 100 (Terman 1916), without mentioning Stern.

7 In this context, Stern explicitly referred to ambitions to select military personnel by use of intelligence tests: “Psychological tests are something else than determining body length or weight, which can only be carried out by non-commissioned officers” (Stern 1912, p. 9).

8 After the First World War, pedagogy was constituted as an independent university discipline, as “humanities pedagogy”. This institutionalization process was characterized by the clear demarcation from empirical psychology.

9 The Republic introduced the common four-year elementary school and decided to abolish the privileged pre-schools.

10 The results of the investigation “contradict those pessimistic views, as if gifted children were unchildlike, unwilling to play, antisocial, and given to ugly tendencies” (Stern 1928a, p. 462).

11 In contrast to Stern, for example, his friend and colleague Otto Lipmann assumed “intelligences”.

12 Stern pointed out forms of giftedness that would today be referred to as “island talents”, and in this context named the gifted and disabled draftsman Gottfried Mint (1768–1814), who had possessed an extraordinary talent for depicting cats and people (Stern 1928a).

13 Only at the end of the 20th and beginning of the 21st century were new approaches in intelligence research developed that contain central elements of Stern’s concept of intelligence and talent. Gardner (1999), Sternberg (2003), Heller et al. (2000), and Weinert (2000), for example, have criticized the measurement of intelligence and the one-sidedness of theories of intelligence that are one-sidedly cognitive.

14 The reason for this gap is certainly the rupture of the First World War and its negative effects on German–American academic relations.

15 From then on, German psychologists oriented themselves towards American psychology, which was scientifically oriented. This also hindered the reception of Stern, who had called for the combination of scientific and philosophical approaches in psychology.
